# Dyskerin Localizes to the Mitotic Apparatus and Is Required for Orderly Mitosis in Human Cells

**DOI:** 10.1371/journal.pone.0080805

**Published:** 2013-11-26

**Authors:** Faizan Alawi, Ping Lin

**Affiliations:** Department of Pathology, School of Dental Medicine, University of Pennsylvania, Philadelphia, Pennsylvania, United States of America; Institut de Génétique et Développement de Rennes, France

## Abstract

Dyskerin is a highly conserved, nucleolar RNA-binding protein with established roles in small nuclear ribonucleoprotein biogenesis, telomerase and telomere maintenance and precursor rRNA processing. Telomerase is functional during S phase and the bulk of rRNA maturation occurs during G_1_ and S phases; both processes are inactivated during mitosis. Yet, we show that during the course of cell cycle progression, human dyskerin expression peaks during G_2_/M in parallel with the upregulation of pro-mitotic factors. Dyskerin redistributed from the nucleolus in interphase cells to the perichromosomal region during prometaphase, metaphase and anaphase. With continued anaphase progression, dyskerin also localized to the cytoplasm within the mid-pole region. Loss of dyskerin function via siRNA-mediated depletion promoted G_2_/M accumulation and this was accompanied by an increased mitotic index and activation of the spindle assembly checkpoint. Live cell imaging further revealed an array of mitotic defects including delayed prometaphase progression, a significantly increased incidence of multi-polar spindles, and anaphase bridges culminating in micronucleus formation. Together, these findings suggest that dyskerin is a highly dynamic protein throughout the cell cycle and increases the repertoire of fundamental cellular processes that are disrupted by absence of its normal function.

## Introduction

Dyskerin is an evolutionarily conserved protein that binds to and stabilizes small non-coding RNAs that are characterized by the H/ACA secondary structure [1]. Through binding to specific H/ACA RNAs, dyskerin plays critical roles in multiple important cellular processes. Most H/ACA small nucleolar RNAs (snoRNAs) direct the pseudouridination and post-transcriptional processing of precursor rRNA [1]. Dyskerin is a pseudouridine synthase and, in a ribonucleoprotein (RNP) complex containing three other conserved proteins, catalyzes the conversion of specific uridine residues to pseudouridines in nascent rRNA. Mouse, yeast, and *Drosophila* dyskerin-null mutants are lethal [2]–[4]. Pseudouridination is severely impaired in these mutants, suggesting the importance of dyskerin-mediated rRNA processing for normal growth and survival.

Dyskerin is also a core component of the telomerase RNP and is required for telomere maintenance [5], [6]. Dyskerin binds to and stabilizes telomerase RNA (TERC) within the complex; TERC harbors an H/ACA domain at its 3′ end. Through binding to H/ACA small Cajal body RNAs, dyskerin is also implicated in the pseudouridination and processing of small spliceosomal RNAs [1]. At least 350 non-coding RNAs with H/ACA box motifs have also been identified but not yet ascribed specific functions [1], [7]. Dyskerin can associate with these RNAs. Thus, it is conceivable that dyskerin may regulate other important cellular functions.

Several lines of evidence indicate a role for dyskerin in potentiating cell proliferation [5], [6], [8], [9]. To this end, dyskerin has an integral function during S phase inasmuch as the telomerase RNP is recruited to telomeres during DNA replication [10]. We previously showed that dyskerin expression is upregulated in experimental conditions that promote cell growth with or without proliferation [11]. In the absence of proliferation cell size increases without DNA replication, thereby also implicating dyskerin in a putative G_1_ function; the bulk of rRNA processing occurs in G_1_ and S [12]. Yet, dyskerin depletion in human cell lines led to G_2_/M accumulation concomitant with an increase in the proportion of multi-polar spindled mitoses relative to control cells [9].

During interphase, dyskerin localizes to the nucleolus, which is the main site of nascent rRNA processing, and to Cajal bodies [1]. These latter sub-nuclear organelles serve as the primary site for spliceosomal RNA processing, and assembly and maturation of small nuclear and snoRNPs, including the telomerase RNP [1], [13]. Cajal bodies are most prominent in cells exhibiting high levels of transcriptional activity; their number and size are greatest at the G_1_/S boundary [13]. The nucleolus and Cajal bodies dissociate early in mitosis and re-form in late mitosis and early in G_1_, respectively [13], [14]. Yet the fate and function of dyskerin throughout cell cycle progression has not been definitively established.

Described herein, we show that dyskerin expression peaks during G_2_/M and that the protein distributes to distinct compartments in mitotic cells. We further demonstrate that loss of dyskerin function has a broadly disruptive effect on mitosis and triggers the spindle-assembly checkpoint. Together this suggests that dyskerin may have important functions throughout the cell cycle and increases the repertoire of fundamental biologic processes affected by absence of its normal function.

## Materials and Methods

### Cell culture

HeLa cervical carcinoma (American Type Culture Collection; Manassas, VA), HeLa-H2B-GFP [15] and UM-SCC1 oral squamous carcinoma cells [16] were grown in Dulbecco's minimal essential medium with GlutaMAX (Invitrogen, Carlsbad, CA) containing 10% fetal bovine serum. OKF6-TERT2 cells [17] were cultured in Defined Keratinocyte-Serum Free Media (Invitrogen) supplemented with 25 µg/ml bovine pituitary extract, 0.2 ng/ml epidermal growth factor, and 0.4 mM CaCl_2_. All media formulations were supplemented with 100 IU/mL penicillin and 100 IU/mL streptomycin (Invitrogen).

For cell synchronization, UM-SCC1 cells were treated with 2.5 mM thymidine (Sigma-Aldrich, St. Louis, MO) for 16 hrs, washed with PBS, cultured for nine hours in normal growth medium and then treated with 2.5 mM thymidine for another 16 hrs. After removing the thymidine, the cells were returned to normal medium and harvested at the indicated times. The cells were maintained at 37°C for the duration of the chemical incubations.

### siRNA transfections

Custom-designed siGENOME SMARTPool siRNA duplexes targeting *DKC1* [9] and pre-designed siGENOME SMARTPool siRNA targeting *MAD2* and a negative control, siGENOME Non-Targeting siRNA pool #2 were obtained from Dharmacon (Lafayette, CO). Briefly, cells at a confluency of 30% to 50% were transfected with the siRNAs using Lipofectamine 2000 (Invitrogen) as per the manufacturer's instructions.

### Protein extraction and immunoblotting

Cells were lysed using M-PER supplemented with HALT Protease Inhibitor Cocktail (Pierce Biotechnology, Rockville, IL). Western blots were performed as previously described [9]. Antibodies against dyskerin (sc-48794), α-tubulin (sc-5286), nucleophosmin (sc-56622), GAPDH (sc-32233), and secondary antibodies were obtained from Santa Cruz Biotechnology (Santa Cruz, CA). Antibodies recognizing MAD2 and native histone H3 were purchased from Cell Signaling Technology (Danvers, MA). Also used were anti-phosphorylated histone H3-serine 10 (Active Motif, Carlsbad, CA) and anti-cyclin B1 (BD Biosciences, San Jose, CA). In most cases, the blots were stripped and re-probed with a different antibody.

### Cell cycle analysis

Cells were washed, fixed with ice cold 80% ethanol, resuspended in 0.5 mL cold PBS and then stained with 10 µg/ml propidium iodide containing 1 mg/ml RNase A (Sigma-Aldrich) for 30 min. To assess mitotic index, the cells were fixed, permeabilized with 0.2% bovine serum albumin and 0.1% Triton X-100 (Sigma-Aldrich) in PBS, then labeled with anti-phosphorylated histone H3-serine 10-Alexa Fluor® 488 conjugate (Cell Signaling Technology) and propidium iodide. The cells were analyzed using a BD™ LSR II flow cytometer (BD Biosciences, Sparks, MD). Data analysis was performed using FlowJo Version 10 (Tree Star, Ashland, OR). All statistical analyses were conducted using Student's t-test.

### Apoptosis assay

The translocation of phosphatidylserine to the outer leaflet of the plasma membrane was analyzed by using the Alexa Fluor® 488 Annexin V/Dead Cell Apoptosis Kit (Invitrogen). Three and six days after HeLa cell transfection, floating and trypsinized cells were collected, washed in cold PBS, and resuspended in binding buffer (10 mM HEPES/NaOH pH 7.4, 140 mM NaCl, 2.5 mM CaCl_2_). After incubation with annexin V and propidium iodide for 15 min, the cells were analyzed by flow cytometry within one hr of staining.

### Indirect immunofluorescence

OKF6-TERT2 grown in 4-well Lab Tek chamber slides (Thermo Scientific, Rochester, NY) were fixed with 4% paraformaldehyde, permeabilized with 0.2% bovine serum albumin and 0.1% Triton X-100 in PBS, and immunolabeled with the appropriate primary antibody overnight at 4°C. After washing, incubation with the appropriate conjugated secondary antibody was performed for 30 mins at room temperature. FITC, and Cy-5 conjugated secondary antibodies were obtained from Jackson ImmunoResearch Laboratories (West Grove, PA). DAPI (4,6-diamidino-2-phenylindole) was used to counterstain DNA. The cells were analyzed using a Nikon A1R Laser Scanning Confocal Microscope (Nikon, Melville, NY). Within a given experiment, all images were acquired under the same lighting conditions and exposure times.

### Live cell imaging and analysis

HeLa-H2B-GFP cells were transfected in 2-well Lab-Tek II chambered coverglass slides (Thermo Scientific). Forty-eight hrs later, the slides were placed in an environmental chamber maintained at 37°C with a humidified atmosphere of 5% CO_2_. Images were then captured on the Nikon A1R microscope every five minutes for up to 16 hrs. Analyses were performed using Nikon Element Advanced Research Software package 3.2 (Nikon) and the images were further processed with Adobe Photoshop CS5 (Adobe Systems, San Jose, CA) using the “auto-color” and/or “auto-contrast” functions. Time-lapse videos were collated and edited using Windows Live Movie Maker, version 2011 (Microsoft Corporation, Redmond, WA). All experiments were performed at least in triplicate.

## Results

### Dyskerin expression peaks during G_2_/M

**Figure 1:**
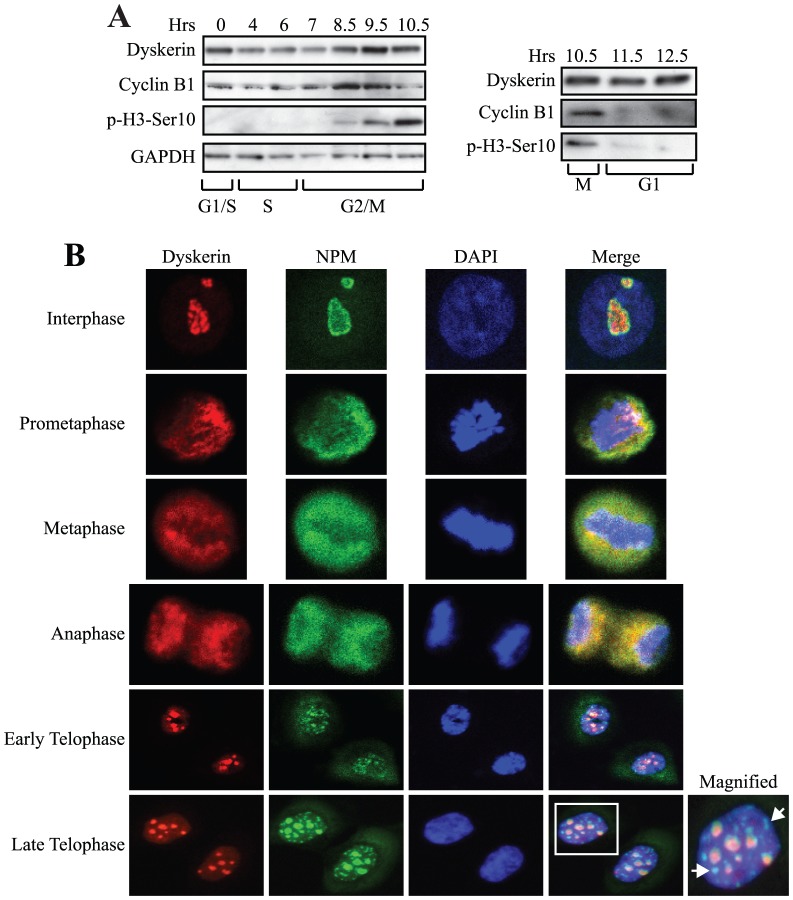
Dyskerin expression peaks during G_2_/M and localizes to distinct compartments throughout mitosis. **A**, UM-SCC1 cells were synchronized at the beginning of S phase using a double thymidine block. Western blot showing expression of dyskerin at various times after release from the block. Cyclin B1 and p-H3-Ser10 expression were used to highlight G_2_/M. GAPDH was used as a loading control. **B**, Dyskerin showed strong co-localization with NPM throughout prometaphase, metaphase and anaphase. Dyskerin partially co-localized with NPM during interphase and telophase. During telophase, nucleoplasmic NPM foci devoid of dyskerin are also seen. The boxed nucleus is magnified to show detail. NPM foci are indicated by arrows. One representative OKF6-TERT2 cell is shown at each mitotic stage.

UM-SCC1 cells were synchronized at the G_1_/S boundary through use of a standard double thymidine block and then released into fresh medium. Dyskerin levels remained essentially unchanged throughout the course of S phase and early G_2_ (Figs. 1A, S1). In late G_2_ (8.5 hours after release from the block), dyskerin expression began to rise before peaking in early mitosis. Dyskerin expression increased in parallel with the upregulation of cyclin B1 and the appearance of phosphorylated histone H3-serine 10 (p-H3-S10). Cyclin B1 is required for mitotic entry and its levels rise and peak in late G_2_/early M phase [18]. Mitotic phosphorylation of histone H3 at Ser 10 also begins to occur in G_2_ but is most robust during metaphase [19]. While cyclin B1 and p-H3-S10 levels decreased in late mitosis, dyskerin expression persisted at relatively high levels, even upon mitotic exit into G_1_. This reinforces the notion that dyskerin may play a functional role during G1. These findings support those of a cDNA microarray study which showed that *DKC1* mRNA levels peak during G_2_/M and remain elevated through G_1_ [20].

### Dyskerin and nucleophosmin (NPM) exhibit a similar distribution in mitotic cells

Next, we investigated the cellular localization of dyskerin in TERT-immortalized primary human oral keratinocytes (OKF6-TERT2). As a control for these experiments, we also examined the localization of NPM. NPM is a highly conserved nucleolar RNA binding protein that plays critical roles in precursor rRNA processing and mitosis [21].

During interphase, dyskerin and NPM were confined to the nucleolus. While the NPM staining pattern was diffuse and smooth throughout the nucleolus, dyskerin expression appeared to be more irregular and less diffuse (Fig. 1B). There was only partial co-localization; this is likely reflective of the distinct compartmentalization of the proteins within the nucleolus [22]. As expected, Cajal bodies were devoid of NPM (not shown). During mitosis, the nuclear membrane and nucleolus are completely disrupted. This was accompanied by re-localization of dyskerin and NPM primarily to the perichromosomal region and to a lesser extent within the cytoplasm during prometaphase and metaphase; both proteins showed an almost identical distribution pattern. During anaphase, dyskerin and NPM also strongly co-localized within the perichromosomal region and the mid-pole cytoplasmic region. It was not until telophase that differences between the two proteins were noted. While both proteins were returned to the nucleus and co-localized in nucleolar organizer regions (NORs), discrete NPM foci were also seen within the nucleoplasm (see late telophase panels, arrows). These foci were devoid of dyskerin. This suggests the possibility that dyskerin may be completely incorporated into NORs prior to NPM. The mitotic localization of dyskerin was identical in all human cell lines examined, including UM-SCC1 and HeLa cells, and in immortalized murine oral keratinocytes (not shown).

### Dyskerin appears to be mostly excluded from the mitotic spindle

**Figure 2:**
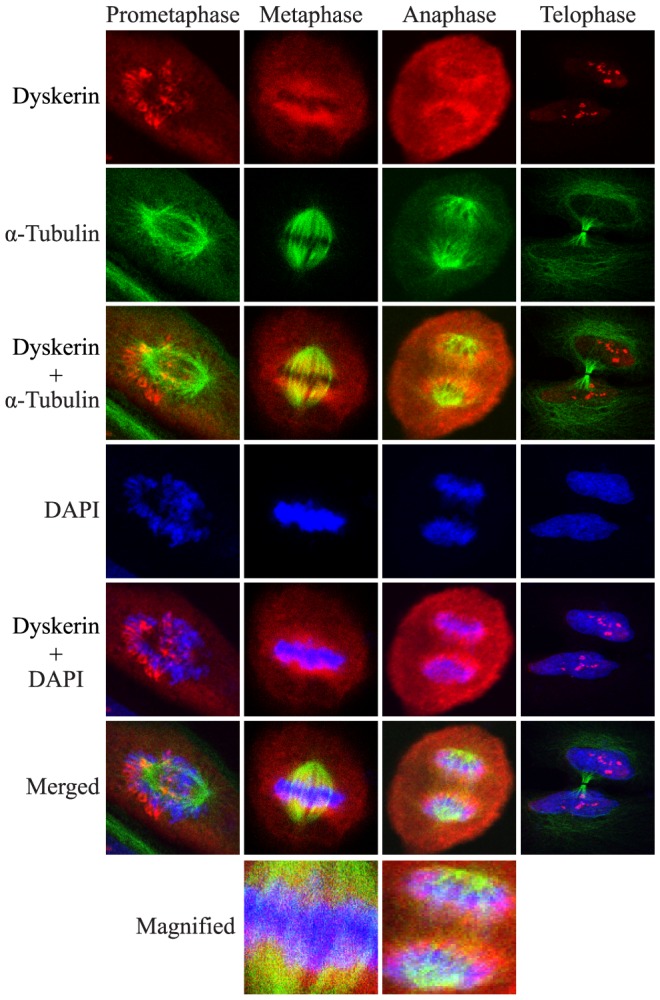
Dyskerin is mostly excluded from the mitotic spindle. Co-localization of dyskerin with α-tubulin was either weak or inconsistently observed. Thus, specific localization of dyskerin with the mitotic spindle could not be reliably ascertained by indirect immunofluorescence. The merged metaphase and anaphase panels are magnified to show detail. One representative OKF6-TERT2 cell is shown at each stage. Dyskerin (red), α-tubulin (green), DAPI (blue).

NPM is associated with the mitotic spindle [21]. Since dyskerin and NPM co-localized throughout much of mitosis, the relationship between dyskerin and the mitotic spindle was assessed. Asynchronous OKF6-TERT2 cells were labeled with antibodies against dyskerin and α-tubulin; the latter is an integral component of the spindle. During prometaphase, dyskerin distributed to the chromosome periphery and showed no co-localization with the burgeoning spindle (Fig. 2). During metaphase and anaphase when the spindle is fully formed, dyskerin remained primarily concentrated in the perichromosomal region and showed only weak and inconsistent co-localization with α-tubulin over several experiments (see magnified panels, and data not shown). This suggests that dyskerin may not be an integral component of the mitotic spindle.

### Loss of dyskerin function increases the mitotic index

**Figure 3:**
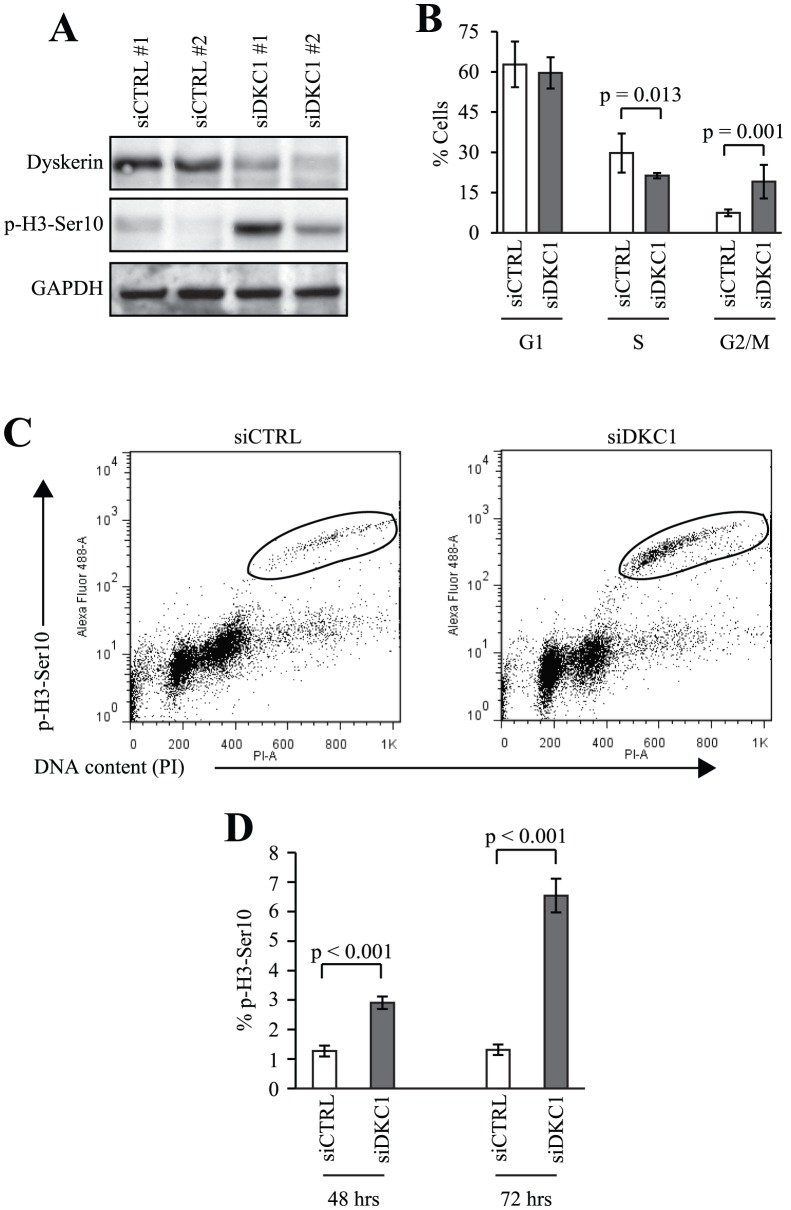
Dyskerin depletion leads to an increased mitotic index. **A**, After 72 hrs, dyskerin levels were decreased in HeLa cells by more than 80% following transfection with two distinct siRNAs directed against *DKC1* (siDKC1) relative to cells transfected with non-target siRNAs (siCTRL). This was accompanied by activation of p-H3-Ser 10 expression. siDKC1 #1 – custom-designed siRNA pool (as described in Materials and Methods), siDKC1 #2 – pre-designed siRNA pool (Dharmacon). **B**, Loss of dyskerin led to a small but statistically significant increase in G_2_/M accumulation as measured by flow cytometry. **C**, The G_2_/M accumulation was partially attributable to an increase in the mitotic fraction of dyskerin-depleted cells 72 hrs after siRNA transfection as measured by anti-p-H3-ser 10-Alexa Fluor® 488 and propidium iodide labeling (circled populations). **D**, The mitotic fractions were quantitated 48 and 72 hrs after siRNA transfection. Experiments in panels B–D were performed using siDKC1 #1.

Next, we sought to define the mitotic phenotype of dyskerin-depleted cells. Using two distinct siRNAs, we achieved an 80–90% decrease in dyskerin expression (siDKC1) as measured by Western blot (Fig. 3A), relative to HeLa cells transfected with a non-specific siRNAs (siCTRL). As we previously demonstrated [9], 72 hrs after transfection dyskerin-depleted cells accumulated in G_2_/M with a corresponding reduction in S phase cells (Fig. 3B). p-H3-S10 staining in conjunction with flow cytometric analysis revealed a 2-fold increase in mitotic siDKC1 cells relative to the siCTRL cells 48 hrs after transfection (p = 0.0005), and a 5-fold increase (p = 0.0001) in mitotic cells 72 hrs after knockdown (Fig. 3C, D). p-H3-S10 protein expression was also increased in whole cell lysates (Fig. 3A).

### Dyskerin depletion disrupts mitotic spindle formation, delays mitotic progression and promotes micronucleus formation

**Figure 4:**
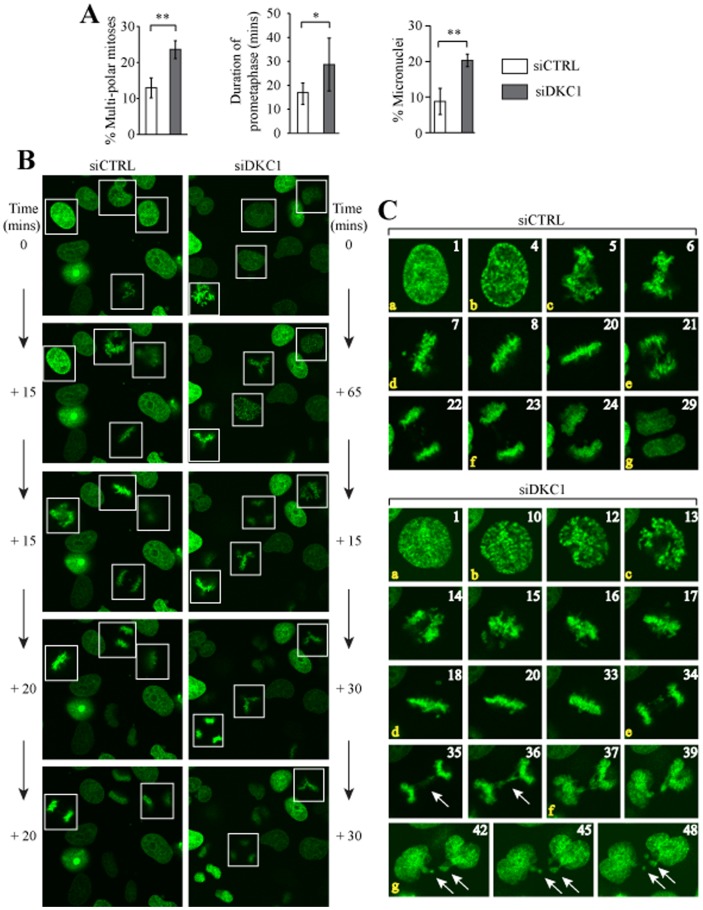
Loss of dyskerin delays prometaphase, disrupts spindle formation and promotes micronucleus formation. HeLa-H2B-GFP cells were transfected with siDKC1 #1 (siDKC1) or control siRNA (siCTRL). After 48 hrs, images were captured in real-time every five minutes for 16 hrs. **A**, Quantitation of multi-polar mitoses as a percentage of the total number of mitoses counted; siCTRL (N = 110), siDKC1 (N = 148). Quantification of prometaphase duration and micronuclei formation; N = 30 for each condition. *p<0.0001, **p<0.002. **B**, Random fields demonstrating four siCTRL and siDKC1 cells undergoing mitosis (white boxes), respectively, are shown for illustrative purposes. The panels at the top are arbitrarily assigned as Time 0. Each panel going down represents the same field and shows the cells as they progressed from prophase through to anaphase. The times listed adjacent to each panel represent the amount of time it took to reach the stage shown, as measured by the number of consecutive image captures multiplied by 5 mins per image capture. All four mitoses in the siDKC1 field yielded tri-polar mitoses. **C**, Single representative cells are shown as they progressed from prophase to cytokinesis. **a**: The panels assigned as ‘1’ represent the cells during prophase. The numbers in the other panels represent the image capture number subsequent to image ‘1’. **b**: siCTRL panel ‘4’ and siDKC1 panel ‘10’ represent late prophase during which the chromatin can be seen condensing. **c**: prometaphase begins; **d**: metaphase begins; **e**: anaphase; **f**: telophase; **g**: cytokinesis. The duration of time needed for this siCTRL cell to progress from b (late prophase) – f (telophase) was 95 mins. The corresponding siDKC1 cell required 130 mins. Lagging chromosomes and a chromatin bridge are seen during anaphase (panels ‘35’ and ‘36’, arrows) in the siDKC1 cell. Micronuclei are also evident following cytokinesis (panels ‘42’-‘48’, arrows).

**Figure 5:**
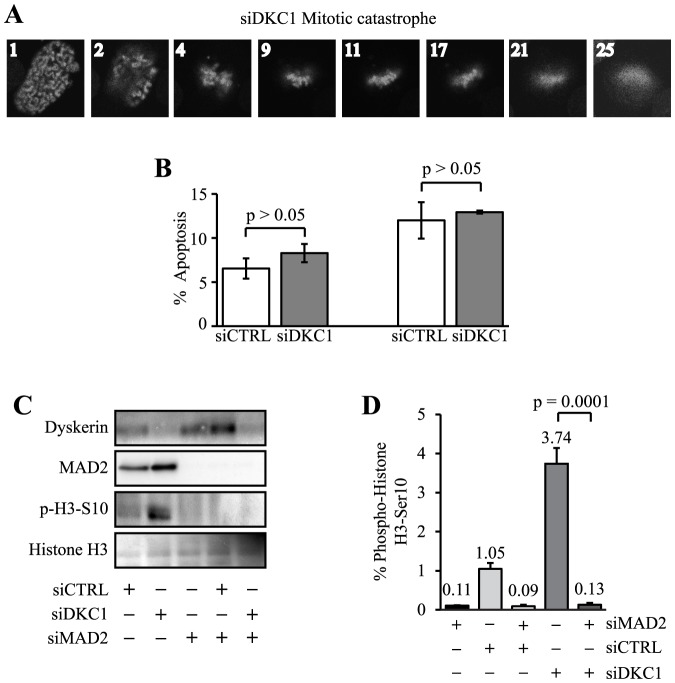
Loss of dyskerin triggers the spindle assembly checkpoint. **A**, Representative Hela-H2B-GFP siDKC1 cell progressing from prophase to mitotic catastrophe. The numbers shown in each panel represent the image capture number subsequent to image ‘1’. **B**, Three and six days after HeLa transfection apoptosis was assessed using Annexin V staining and flow cytometry. Error bars denote the standard deviations derived from three independent transfections at each time point. One representative experiment is shown. **C**, HeLa cells were transfected with CTRL, *DKC1* and/or *MAD2* siRNAs and harvested 72 hrs later. Immunoblot analysis showed almost complete absence of MAD2 and p-H3-Ser 10 expression in MAD2- depleted cells. **D**, In parallel, the cells were fixed and the mitotic fractions were assessed by flow cytometry.

We previously reported that loss of dyskerin function in HeLa cells was associated with a significantly increased incidence of mitoses with atypical mitotic spindles [9]. A similar finding was observed in the current studies using dyskerin-depleted HeLa-H2B-GFP cells (Fig. 4A, B). Time-lapse imaging showed that loss of dyskerin also triggered additional mitotic defects (Videos S1 and S2). In particular, prometaphase was significantly prolonged (p<0.0001) in dividing siDKC1 cells, averaging 28.7±11.1 mins (high – 55 mins, low – 15 mins; median 30 mins) versus 17.0±5.0 mins (high – 25 mins, low – 10 mins; median 15 mins) for the control cells (Fig. 4A, C). Although siDKC1 cells also exhibited a prolonged metaphase, we were unable to reliably quantitate metaphase duration because many of the cells eventually underwent mitotic catastrophe prior to anaphase (Fig. 5A). For cells that successfully reached anaphase, there was no significant difference in anaphase duration between the siDKC1 and siCTRL cells (not shown). However, lagging chromosomes and bridges were more commonly observed in siDKC1 anaphases (Fig. 4C, panels ‘35’, ‘36’). This resulted in a significantly greater of proportion of siDKC1 mitoses eventuating in micronuclei relative to the siCTRL cells (p = 0.0013; Fig. 4A, C panels ‘42’, ‘45’, ‘48’).

### Loss of dyskerin triggers the spindle-assembly checkpoint

Via time-lapse imaging, we also noted that a number of siDKC1 cells underwent spontaneous degeneration during the course of mitosis (Fig. 5A). Loss of dyskerin did not induce apoptosis of HeLa cells (Fig. 5B). Thus, these abortive mitoses in conjunction with a delay in mitotic progression led us to speculate that loss of dyskerin may have triggered activation of the spindle assembly checkpoint (SAC).

The SAC is an evolutionarily-conserved surveillance mechanism that ensures progression to anaphase only when all chromosomes are appropriately attached to the mitotic spindle and aligned within the metaphase plate [23]. MAD2 is a core component of the SAC. Co-depletion of dyskerin and MAD2 expression almost completely abrogated the mitotic cell accumulation induced by dyskerin-depletion alone, as measured by p-H3-S10 expression using Western blot (Fig. 5C) and flow cytometry (Fig. 5D). Together, these data suggest that loss of dyskerin function triggered an array of mitotic defects accompanied by activation of the SAC.

## Discussion

An array of nucleolar proteins required for critical interphase functions localize to the perichromosomal region during mitosis. This includes various rRNA processing factors, such as NPM (as observed herein), nucleolin, fibrillarin, BOP1, and PES1 [14], [21], [24]–[29]. Of note is that each of the aforementioned proteins has also been associated with the mitotic spindle [21], [24]–[27], [30], [31]. We did not observe consistent co-localization of dyskerin with α-tubulin. Nonetheless, using mass spectrometry, dyskerin was previously identified in mitotic spindle extracts derived from plant, yeast, *Drosophila*, *Xenopus*, and human HeLa cells [30]–[34], and yeast dyskerin (Cbf5p) was initially described as a microtubule-binding protein [35]. While further study is needed to definitively establish an association between dyskerin and the mitotic spindle, our findings lead us to conclude that the perichromosomal localization of dyskerin is specific and not an experimental artifact.

Partially processed pre-rRNA and mature 18S and 28S rRNA are also preserved in mitotic cells and enriched within the perichromosomal region during metaphase and anaphase [36]. Yet, in mammalian cells, rRNA processing diminishes during G_2_ and completely ceases early in mitosis, only to be restored upon mitotic exit [12]. Similar to the effects of dyskerin depletion, loss of function of NPM, nucleolin, PES1, and BOP1, respectively, induces mitotic spindle defects, anaphase bridges, a propensity for micronucleus formation, delayed mitotic progression and, where studied, activation of the SAC [21], [24]–[27]. Thus, it is intriguing to speculate that dyskerin and other rRNA processing factors may have evolutionarily conserved functions during mitosis that are independent of their respective roles in rRNA maturation.

Like rRNA processing, telomerase is also inactivated during mitosis [10]. However, telomere maintenance is required throughout the cell cycle. While the function of the perichromosomal region remains poorly understood, it may contribute to preservation of chromosome structure and integrity and ensure equal distribution of DNA between daughter cells [14], [29]. To this end, the perichromosomal region also harbors several telomere maintenance proteins [14], [29].

During S phase, dyskerin contributes to telomere maintenance by stabilizing TERC within the telomerase complex [5]. Constitutive loss of dyskerin function decreases TERC steady-state levels thereby disrupting normal telomerase function [5], [6]. Over time, decreased telomerase activity leads to telomere shortening, and excessive telomere shortening may lead to fusion of chromosome ends and the formation of anaphase bridges [37], [38] similar to those we observed in our experiments.

Within 48 hrs after *DKC1* siRNA transfection in telomerase-positive HeLa and UM-SCC1 cells, TERC expression is decreased by less than 50% relative to controls (ref [9] and unpublished data). In our current studies, the mitotic index of siDKC1 cells had more than doubled only 48 hrs after transfection. We also observed anaphase bridges and an array of other mitotic defects in dyskerin-depleted cells less than 65 hrs after siRNA transfection. Although this was a relatively short period of time, we cannot exclude the possibility that telomere lengths were sufficiently shortened to the extent that they could have contributed to the observed mitotic phenotypes. Alternatively, as Gu et al [6] recently described in murine cells, it is possible that loss of human dyskerin function may also disrupt telomere homeostasis independently of telomere length regulation. Our findings suggest a possible role for dyskerin in regulating mitotic progression. It will be important to establish whether this represents a novel function for dyskerin or if this is a newly recognized complication resulting from a disturbance in cellular processes and/or molecular interactions already known to be dependent upon normal dyskerin function. Dyskerin's role in several critical cellular processes is presumed to be dependent upon its binding to H/ACA RNAs [1]. Whether dyskerin is bound to H/ACA RNA, other proteins and/or directly to the chromosomal DNA during mitosis will require more detailed study.

Finally, germline mutations in *DKC1* give rise to X-linked dyskeratosis congenita (DC) [2], [5]. X-linked DC is associated with oral leukoplakia, poikiloderma, aplastic anemia, premature aging and increased susceptibility to oral squamous cell carcinoma and other epithelial and lymphoreticular cancers [39]. The clinical manifestations of this unusual disorder have been mainly attributed to premature telomere attrition [5], [6], [39]. As noted above, excessive telomere shortening promotes anaphase bridges and breakage of these bridges can lead to micronucleus formation [37], [38]. Studies suggest that increased micronucleus formation may be associated with advanced age, enhance cancer susceptibility and impart an increased risk for an array of other systemic disorders [40]–[42]. X-linked DC is generally associated with a more diverse and severe phenotype and a poorer prognosis than some other genetic forms of DC, particularly those caused by defects in other telomerase RNP components [39]. Moreover, while X-linked DC has been characterized as a chromosomal instability syndrome [43], the mechanisms by which cancer susceptibility is increased in this disorder remain unclear [44].

An increasing body of evidence suggests that telomere dysfunction may not completely account for the phenotype of X-linked DC [2], [6], [44]–[46]. Although a disruption in rRNA processing is clearly evident in mouse models of X-linked DC [2], [6], a similar impairment is not readily apparent in dyskerin-mutant human cells [5], [44]. Dokal et al [43] analyzed metaphase spreads of peripheral blood lymphocytes derived from patients with X-linked DC and identified a single spontaneous tripolar metaphase out of 30 metaphases. Skin fibroblast cultures from a female patient with dyskeratosis congenita of unknown genetic subtype revealed various mitotic disturbances, including anaphase bridges and micronuclei [47]. To our knowledge the prevalence of micronuclei in X-linked DC cells has not been assessed. Hence, it will be interesting to investigate whether mitotic defects may contribute to the pathogenesis and phenotype of human X-linked DC.

## Supporting Information

Figure S1 **Cell cycle profiles after release of UM-SCC1 cells from double thymidine block.** UM-SCC1 cells entered G_2_ by 7 hrs after release from the thymidine block and peaked in G_2_/M between 8.5–10.5 hrs. Transit into G_1_ was evident by 11.5 hrs. These experiments were performed in parallel to those shown in Fig. 1A.
(PDF)

Video S1 **Time lapsed live-cell imaging of HeLa-H2B-GFP cells transfected with siDKC1.**
(WMV)

Video S2 **Time lapsed live-cell imaging of HeLa-H2B-GFP cells transfected with siCTRL.**
(WMV)
