# Supplementary figures and images for: Dyskerin Localizes to the Mitotic Apparatus and Is Required for Orderly Mitosis in Human Cells
Source: PLoS One. 2013 Nov 26;8(11):e80805. doi: 10.1371/journal.pone.0080805 (PMC3841160; doi:10.1371/journal.pone.0080805)

Supp. Figure S1

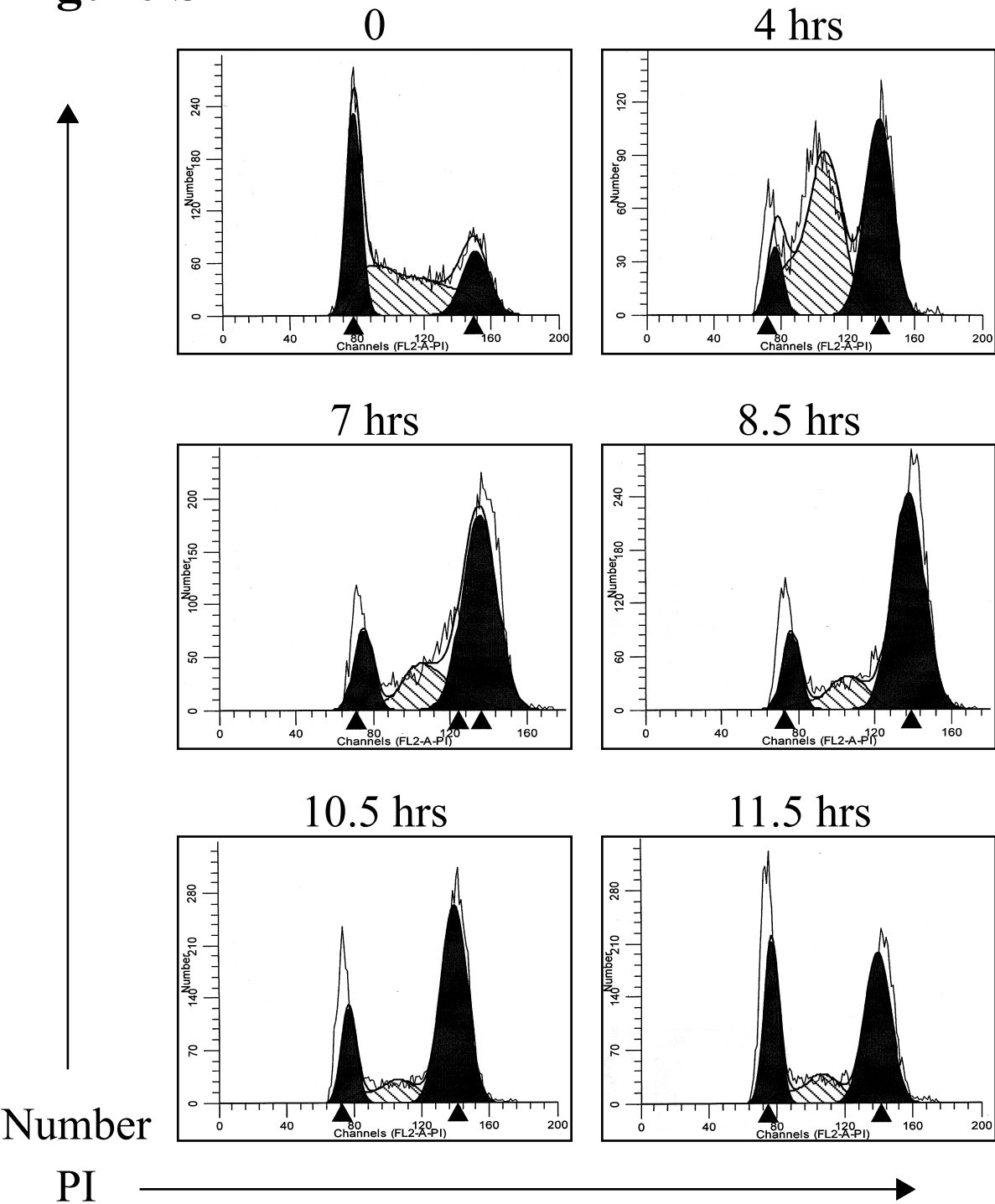

| Time (hrs) | G <sub>1</sub> (%) | S (%) | G <sub>2</sub> /M (%) |
|------------|--------------------|-------|-----------------------|
| 0          | 34.3               | 44.6  | 21.1                  |
| 4          | 11.6               | 52.9  | 35.4                  |
| 6          | 8.0                | 50.1  | 41.9                  |
| 7          | 14.3               | 22.5  | 63.2                  |
| 8.5        | 13.9               | 14.5  | 71.6                  |
| 10.5       | 18.0               | 13.1  | 68.9                  |
| 11.5       | 27.6               | 15.9  | 56.5                  |
| 12.5       | 31.4               | 15.9  | 52.7                  |

Supplement: Figure S1 — Cell cycle profiles after release of UM-SCC1 cells from double thymidine block. UM-SCC1 cells entered G2 by 7 hrs after release from the thymidine block and peaked in G2/M between 8.5–10.5 hrs. Transit into G1 was evident by 11.5 hrs. These experiments were performed in parallel to those shown in Fig. 1A. (PDF) [file pone.0080805.s001.pdf]
